# A tetravalent dengue nanoparticle stimulates antibody production in mice

**DOI:** 10.1186/1477-3155-10-13

**Published:** 2012-03-22

**Authors:** Elisângela F Silva, Mariana Orsi, Ângela L Andrade, Rosana Z Domingues, Breno M Silva, Helena RC de Araújo, Paulo FP Pimenta, Michael S Diamond, Eliseu SO Rocha, Erna G Kroon, Luiz CC Malaquias, Luiz FL Coelho

**Affiliations:** 1 Institute of Biomedical Science, Federal University of Alfenas, Minas Gerais, Brazil; 2 Department of Chemistry, Federal University of Ouro Preto, Ouro Preto, Minas Gerais, Brazil; 3 Department of Chemistry, Federal University of Minas Gerais, Belo Horizonte, Minas Gerais, Brazil; 4 Department of Biological Sciences, Federal University of Ouro Preto, Ouro Preto, Minas Gerais, Brazil; 5 Laboratory of Medical Entomology, Instituto René Rachou, Fundação Oswaldo Cruz, Belo Horizonte, Minas Gerais, Brazil; 6 Departments of Medicine, Molecular Microbiology, Pathology & Immunology, Washington University School of Medicine, Saint Louis, Missouri, USA; 7 Virus Laboratory, Department of Microbiology, Institute of Biological Sciences, Federal University of Minas Gerais, Belo Horizonte, MG, Brazil

**Keywords:** inactivated Dengue vírus, Nanoparticles, humoral response

## Abstract

**Background:**

Dengue is a major public health problem worldwide, especially in the tropical and subtropical regions of the world. Infection with a single *Dengue virus *(DENV) serotype causes a mild, self-limiting febrile illness called dengue fever. However, a subset of patients experiencing secondary infection with a different serotype progresses to the severe form of the disease, dengue hemorrhagic fever/dengue shock syndrome. Currently, there are no licensed vaccines or antiviral drugs to prevent or treat dengue infections. Biodegradable nanoparticles coated with proteins represent a promising method for in vivo delivery of vaccines.

**Findings:**

Here, we used a murine model to evaluate the IgG production after administration of inactivated DENV corresponding to all four serotypes adsorbed to bovine serum albumin nanoparticles. This formulation induced a production of anti-DENV IgG antibodies (p < 0.001). However, plaque reduction neutralization assays with the four DENV serotypes revealed that these antibodies have no neutralizing activity in the dilutions tested.

**Conclusions:**

Our results show that while the nanoparticle system induces humoral responses against DENV, further investigation with different DENV antigens will be required to improve immunogenicity, epitope specicity, and functional activity to make this platform a viable option for DENV vaccines.

## Background

*Dengue virus *(DENV) is a major public health problem worldwide, especially in the tropical and subtropical areas with around 2.5 billion people living in areas at risk [1]. The disease is caused by a positive sense, single-stranded RNA virus that belongs to genus *Flavivirus*, family *Flaviviridae*. DENV is transmitted to humans primarily after a bite by an infected *Aedes aegypti *and *Aedes albopictus *mosquitoes. Infection with one of the DENV serotypes (DENV-1, -2, -3 and -4) causes a mild, self-limiting febrile illness called dengue fever (DF). However, after secondary infection, a small subset (~0.5%) develop the dengue hemorrhagic fever (DHF)/dengue shock syndrome (DSS), the severe form of the disease [2].

While vaccines could potentially prevent DENV infection or disease in humans, none are currently licensed despite decades of intensive research [3]. To date, several approaches have been developed towards generating a tetravalent anti-DENV vaccine including live-attenuated strains, inactivated strains, subunit DNA or plasmid vaccines, and recombinant proteins [4]. Our group has begun vaccine studies using a unique platform, the nanoparticles. Biodegradable nanoparticles are currently used as drug carriers or as adjuvants for vaccines [5]. Polymeric nanoparticles with adsorbed or entrapped antigens represent a novel method for controlling the release of immunogens and to optimizing the immune response via selective targeting of the antigen presenting cells [6]. In this exploratory study we evaluated the anti-DENV IgG response in mice immunized with bovine serum albumin nanoparticles adsorbed with all four serotypes of inactivated DENV.

## Methods

### Cell culture and virus production

C6/36 *Aedes albopictus *cells were grown in L-15 medium (Cultilab, Brazil) supplemented with 10% (v/v) heat-inactivated fetal bovine serum (FBS) (Cultilab, Brazil), 100 μg/mL penicillin and 100 μg/mL streptomycin at 28°C. The DENV-1, 2, 3 and 4 were isolated from dengue infected patients in Brazil and were kindly donated by Dr. Erna G. Kroon (Lab Virus, Federal University of Minas Gerais, Brazil). The propagation of each serotype of DENV was carried out in separate C6/36 cell cultures flasks. The cells were infected with DENV-1, 2, 3 or 4 at a multiplicity of infection (MOI) of 0.1 and incubated at 28°C for a week. After the development of cell syncytia, the supernatants were harvested, and titrated by standard plaque assay in LLC-MK2 cells [7]. Heat-inactivated virus was prepared by incubating virus samples in a 55°C water bath for one hour as described previously [8].

### Nanoparticle preparation and characterization

The nanoparticles were obtained by the addition of ethanol dropwise (ethanol:water relation 1,5:1) to an aqueous solution of bovine serum albumin (BSA) (2% w/v). The coacervates were hardened by adding 50 μL of 25% glutaraldehyde while stirring for 2 hours at room temperature. The BSA-nanoparticles were purified by three cycles of centrifugation at 13,000 g for 30 minutes to eliminate free BSA and the excess of the crosslinking agent. The supernatants were removed and the pellets resuspended in sterile PBS (final concentration of 20 mg/mL). For adsorption of inactivated viral particles to the surface of the nanoparticles (NP+DENV), 1 mL suspension of the tetravalent DENV antigenic suspension (equivalent of 1. 2 × 10^4 ^plaque forming units (PFU) for each serotype) was incubated with 1 mL of NP at 20 mg/mL. After rapid homogenization (30 seconds), the nanoparticles were purified by three successive centrifugations, each at 13,000 g for 30 minutes, 20°C. The supernatants were collected after each centrifugation and tested by Bradford assay [9]. To determine the amount of protein adsorbed to the nanoparticles, a colorimetric assay was used and compared to the input protein and that recovered in the supernatants after centrifugation.

The presence of viral particles on the surface of NPs was demonstrated by ELISA assays using the NP+DENV as antigen. Briefly, 96-well microtiter plates were coated with NP+DENV (200 μg/well) in carbonate buffer, incubated overnight at 4°C and then washed with PBS containing 0.05% Tween-20 (PBST). Subsequently, the plates were blocked with 100 μl/well of blocking buffer (5% dry milk in PBST) for 2 h at 37°C. After three washes, 100 μl of monoclonal anti-DENV-1 (E95), anti-DENV-2 (E96), anti-DENV-3 (E51) or DENV-4 (E88) was added at 1 μg/mL [10-12] in triplicate and incubated for 1 h at 37°C. After three washes, the plates were incubated for 1 hour at 37°C with 100 μl/well of horseradish-peroxidase (HRP)-labeled anti-mouse IgG (Sigma) diluted 1:5000 in PBST. After three additional washes, the plates were incubated for 15 minutes with 0.1 ml of 0.045% H_2_O_2 _and 0.4 mg/ml of o-phenylenediaminedihydrochloride (OPD) in phosphate-citrate buffer (0.1 M citric acid, 0.2 M sodium phosphate dibasic, pH 5.0). The reaction was stopped by adding 50 μl of 2 M H_2_SO_4_. The optical densities were read at 450 nm with a microplate reader. The zeta potential of the NPs were determined with a Zetasizer 3000 HS (Malvern Instruments, UK). The surface morphology and the diameter of the formulated nanoparticles were visualized by scanning electron microscopy (Model JEOL 560).

### Immunization protocol and quantification of anti-DENV and anti-BSA IgG

Six-week-old male Swiss Webster mice were used for immunizations. All mice were maintained with free access to sterile food and water, and protocols were approved by the local Animal Experimentation Ethics Committee. Mice were immunized via the subcutaneous route using 100 μg of NP (n = 15) or NP+DENV (n = 20) on days 0, 7 and 14. The mice were sacrificed 7 days after the last immunization (at day 21). Additional mice were immunized with PBS (negative control) (n = 10) or 1.2 × 10^4 ^PFU of the tetravalent DENV heat-inactivated antigenic suspension (positive control) (n = 10). Blood was collected by cardiac puncture and sera were separated for ELISA. To perform IgG quantification, 96-well microtiter plates were coated with 500 PFU/well of the inactivated DENV particles (serotypes 1 to 4) in carbonate buffer or BSA (2 μg/well). An ELISA was performed exactly as described above for quantitation of viral particles on NP, except virions were captured directly and sera from mice were used as the primary antibody. An analysis of ELISA results was performed using analysis of variance (ANOVA), and the differences between groups were evaluated with Tukey's test.

### Plaque Reduction Neutralization assay (PRNA)

Briefly, heat-inactivated mouse sera were serially diluted two-fold from 1:20 to 1:320 in media and then mixed with a single DENV serotype (20 to 50 PFU/well) and incubated for 1 hour at 37°C. The mixture was inoculated onto LLC-MK2 cells in 24-well plates. Plates were incubated for 1 h before media was aspirated and replaced with 0.5 mL of 0.8% carboximethylcellulose medium (with 2% FBS). The plates are incubated at 37°C (5% CO2) for five days. At that time, cells were stained with a crystal violet solution (0.5% crystal violet, 10% ethanol and 1% paraformaldehyde) for 20 minutes, washed again, and the viral plaques were counted. The positive-control serum sample was a serum sample derived from a human patient and the antibody titers were 1:40 for DENV-1 and 1:20 for DENV-2, 3 and 4. Experiments were performed in duplicate.

## Results and discussion

The development of a dengue vaccine is considered challenging, because it must provide protective immunity against the four serotypes, without causing adverse side effects associated with incomplete immunity against a single serotype. Although the pathogenesis of the disease and protection mechanisms are not fully understood, the production of neutralizing antibodies is believed to be essential to the effectiveness of a DENV vaccine [13]. Biodegradable nanoparticles made by BSA coated with proteins represent a promising method for in vivo delivery of protein vaccines directly to the immune system.

**Figure 1:**
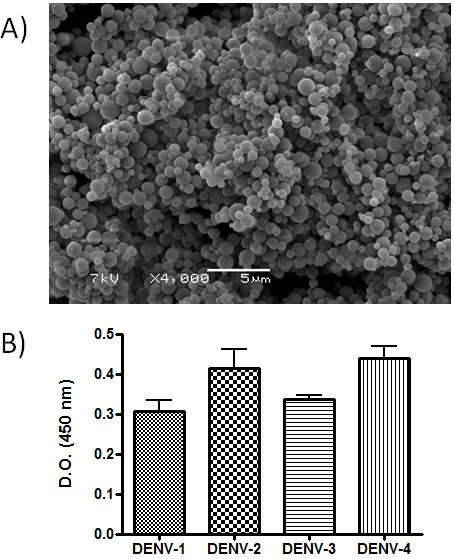
**Nanoparticle characterization**. A) Scanning electronic microscopy of the BSA nanoparticles. B) Adsorption of DENV onto the surface of BSA nanoparticles. BSA nanoparticles were incubated with the tetravalent solution of inactivated DENV and later purified by three cycles of centrifugation. The presence of viral particles on the surface of NPs was demonstrated by ELISA assays using monoclonal antibodies against DENV-1, 2, 3 or 4. Bars represent the mean of data collected from three independent experiments.

The resulting BSA nanoparticles had a mean diameter of 686.9 nm and the mean zeta potential of 35.8 mV. Scanning electron micrograph images of the nanoparticles revealed their regular spherical shape (Figure 1A). The BSA nanoparticle adsorbed 28.8 ± 6.2% of the total proteins present in the tetravalent DENV antigenic suspension, and the presence of the inactivated viral particles on the surface of the NPs was demonstrated by a conventional ELISA assay using monoclonal antibodies against all four DENV serotypes. As shown (Figure 1B), all four DENV serotypes were detected on the surface of nanoparticles. The adsorption process could be attributed to the force of attraction between virions and BSA. Analogously, hepatitis B virus is able to interact with albumin via its surface proteins, suggesting that the same mechanism could occur between NPs BSA and inactivated DENV viral particles [14].

**Figure 2:**
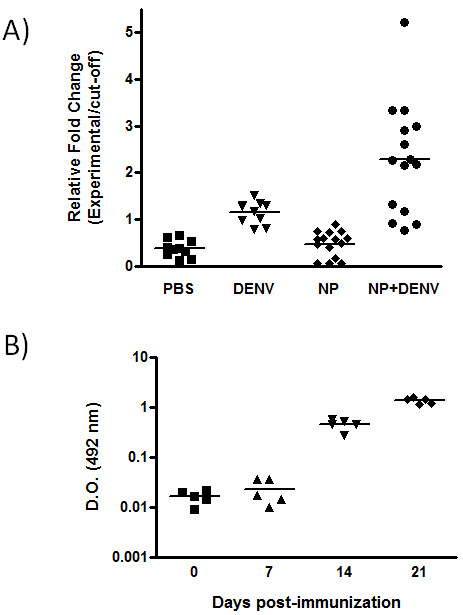
**Antibody production in mice immunized with NP+DENV**. A) Swiss mice were vaccinated according to the indicated schedule and the sera were collected 1 week after the last immunization. Data presented are values for individual mice and the bar represents the group mean. P < 0.001: PBS versus NP+DENV; DENV versus NP+DENV and NP versus NP+DENV; P < 0.01: PBS versus DENV and DENV versus NP by one-way analysis of variance (ANOVA) followed by Tukey's multiple comparison test. B) Kinetics of IgG production in mice immunized with NP+DENV. The serum were collected on day 0, 7, 14 and 21 post-immunization. Data presented are values for individual mice and the bar represents the group mean. P < 0.01: 0 versus 21 and 7 versus 21 by one-way analysis of variance (ANOVA) followed by Tukey's multiple comparison test.

To investigate if this formulation induced antibody production, groups of outbred Swiss Webster mice were inoculated with 100 μg of NP+DNV per mouse, 1, 2 or 3 times, at one week intervals. As a control group, mice were inoculated with an empty NP (naive group) or with 1.2 × 10^4 ^PFU of tetravalent DENV antigenic heat-inactivated suspension (positive control). We observed a robust production of anti-DENV IgG antibodies (*P *< 0.001) in the mice immunized with NP+DENV (Figure 2A). In contrast, the empty NP or NP+DENV failed to induce an anti-BSA antibody response, confirming the relatively poor immunogenicity of BSA. Despite a relatively high level of anti-DENV antibodies in the immunized mice (NP+DENV), the PRNT showed an absence (PRNA_50 _< 1/20) of neutralizing activity in the serum of these animals in all dilutions tested (data not showed), although we cannot rule out the possibility of some neutralizing activity in dilutions below 1:20. Recent studies indicate that the antibody response to DENV infection consists of a minor population of strongly neutralizing antibody and a major population of cross-reactive, non-neutralizing antibodies (both prM and E) with potential for enhancement of virus infection and disease [15-17]. However, if a high concentration of all DENV serotypes (above 1.2 × 10^4 ^pfu/mL) was used in the adsorption step or a larger concentration of NP+DENV was used in this study, these antibodies could have a neutralizing activity. As nanoparticles are phagocytosed by dendritic cells and other antigen-presenting cells (APCs), the adjuvant effect of BSA-nanoparticles could be explained by their rapid internalization by the skin-resident APCs, such as dendritic cells (DC). The interactions of nanoparticles and DCs also could be facilitated by the presence of the envelope protein of DENV that was derived in insect cells, and thus displays high mannose carbohydrates. The DENV E protein is the only glycoprotein exposed on the surface of mature DENV virions and is responsible for attachment to the host cell surface and also plays an important role in viral entry [18]. Studies have shown that this protein binds to the receptors DC-SIGN (CD209) and the mannose receptor and facilitates the infection of myeloid cells [18-21]. Another factor that may contribute to the increased production of IgG anti-DENV is innate immune signaling via toll like receptor 7 (TLR7). Inactivated viral particles present on the surface of the NP have single-stranded RNA, which can activate TLR7 to induce the synthesis of pro-inflammatory cytokines [22] that enhances antibody responses. A similar mechanism was described by Geeraedts and colleagues using whole inactivated influenza virus. They demonstrate that the viral RNA present in inactivated virus particles could trigger TLR7 to augment the adaptive humoral responses [23].

## Conclusions

Thus, nanoparticles can induce anti-DENV antibodies readily, although further studies in antigen formulation will be required to induce the production of neutralizing antibodies against all four DENV serotypes.

## Competing interests

The authors declare that they have no competing interests.

## Authors' contributions

EFS, MO, LCCM, BMS and LFLC conceived and designed the method, performed the experiments and interpreted the data. ALA and RZD performed the zeta analysis. HRCA and PFPP perfomed the SEM analysis. MSD developed the monoclonal antibodies used in ELISA assay. ESOR and EGK performed the PRNT. LFLC prepared the manuscript. LFLC, MSD and EFS critically revised the content of the manuscript and gave the final approval of the version to be published. All authors read and approved the final manuscript.
